# Significant Enhancement of Circular Polarization in Light Emission through Controlling Helical Pitches of Semiconductor Nanohelices

**DOI:** 10.1021/acsnano.3c07663

**Published:** 2023-10-05

**Authors:** Ziyue Ni, Ping Qin, Hongshuai Liu, Jiafei Chen, Siyuan Cai, Wenying Tang, Hui Xiao, Chen Wang, Geping Qu, Chao Lin, Zhiyong Fan, Zong-Xiang Xu, Guixin Li, Zhifeng Huang

**Affiliations:** † Department of Physics, Hong Kong Baptist University, Kowloon Tong, Kowloon, Hong Kong SAR 999077, People’s Republic of China; ‡ Department of Biology, Hong Kong Baptist University, Kowloon Tong, Kowloon, Hong Kong SAR 999077, People’s Republic of China; § School of Science, Harbin Institute of Technology, Shenzhen 518055, People’s Republic of China; ∥ Department of Materials Science and Engineering, Southern University of Science and Technology, Shenzhen, Guangdong 518055, People’s Republic of China; ⊥ Department of Chemistry, Southern University of Science and Technology, Shenzhen, Guangdong 518055, People’s Republic of China; # Department of Electronic and Computer Engineering, The Hong Kong University of Science and Technology, Clear Water Bay, Kowloon, Hong Kong SAR 999077, People’s Republic of China; ∇ Department of Chemistry, Guangdong-Hong Kong-Macao Joint Laboratory for Photonic-Thermal-Electrical Energy Materials and Devices, Southern University of Science and Technology, Shenzhen, Guangdong 518055, People’s Republic of China; ○ School of Chemistry and Chemical Engineering, Harbin Institute of Technology, Harbin 150001, People’s Republic of China; ◆ Department of Physics, The Chinese University of Hong Kong, Shatin, New Territories, Hong Kong SAR 999077, People’s Republic of China; ◇ Department of Chemistry, The Chinese University of Hong Kong, Shatin, New Territories, Hong Kong SAR 999077, People’s Republic of China

**Keywords:** semiconductor nanohelices, circularly polarized light emission, circularly polarized luminescence, circularly polarized scattering, glancing angle deposition

## Abstract

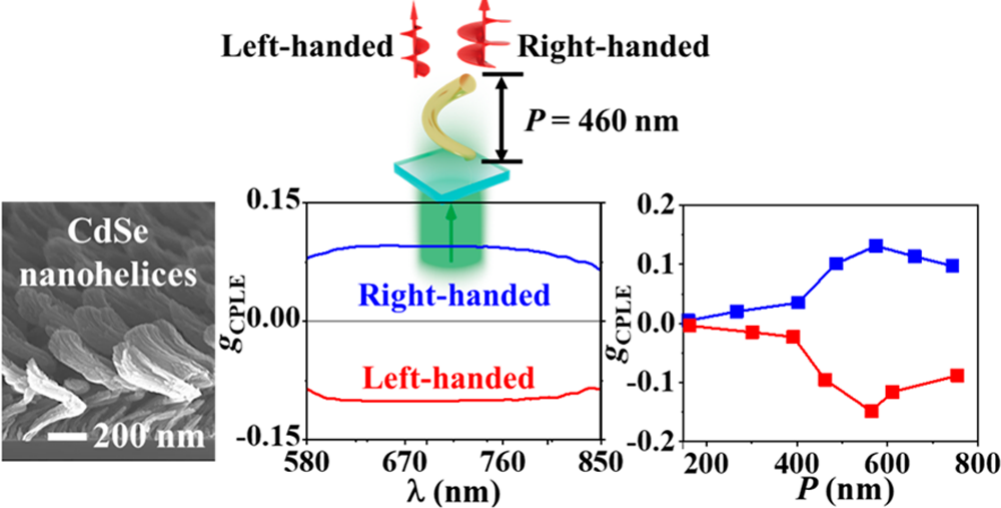

Circularly polarized light emission (CPLE) can be potentially applied to three-dimensional displays, information storage, and biometry. However, these applications are practically limited by a low purity of circular polarization, i.e., the small optical dissymmetry factor *g*_CPLE_. Herein, glancing angle deposition (GLAD) is performed to produce inorganic nanohelices (NHs) to generate CPLE with large *g*_CPLE_ values. CdSe NHs emit red CPLE with *g*_CPLE_ = 0.15 at a helical pitch (*P*) ≈ 570 nm, having a 40-fold amplification of *g*_CPLE_ compared to that at *P* ≈ 160 nm. Ceria NHs emit ultraviolet–blue CPLE with *g*_CPLE_ ≈ 0.06 at *P* ≈ 830 nm, with a 10^3^-fold amplification compared to that at *P* ≈ 110 nm. Both the photoluminescence and scattering among the close-packed NHs complicatedly account for the large *g*_CPLE_ values, as revealed by the numerical simulations. The GLAD-based NH-fabrication platform is devised to generate CPLE with engineerable color and large *g*_CPLE_ = 10^–2^–10^–1^, shedding light on the commercialization of CPLE devices.

Circularly polarized light emission (CPLE), denoted as the differential emission of left- and right-handed circularly polarized light (LCP and RCP, respectively), has attracted increasing attention, owing to promising applications in the fields of three-dimensional displays,^1^ bioimaging,^2^ biosensing,^3,4^ and information storage.^5^ Various chiral materials and structures have been fabricated to produce CPLE in the ultraviolet (UV)–visible–near-infrared (NIR) region. Typically, CPLE is attributed to circularly polarized luminescence (CPL, denoted as the differential emission of LCP and RCP luminescence) through an emission from a chiral excited state of chiral materials with a size much smaller than the excitation wavelength (or a subwavelength size), such as chiral molecules with chromophores,^6^ metal–organic complexes,^7^ semiconductor quantum dots modified with chiral ligands,^8^ conjugated polymers,^9,10^ supramolecules,^11,12^ and two-dimensional perovskites with chiral organic cations.^1^ When their sizes are comparable to the excitation wavelength (such as assemblies of chiral nanoclusters,^2^ superstructures with multiscale chirality,^3^ chiral template-induced nanoassemblies,^5,13^ chiral nanocomposites,^14^ and chiral composites with a photonic band gap^15^), CPLE has an additional or even dominant contribution from circularly polarized scattering (CPS),^16^ denoted as the preferential scattering of LCP or RCP.^17^

It is of essential importance for circular polarization optics to generate either LCP or RCP emission, and the degree of circular polarization has been quantitatively evaluated in terms of the optical dissymmetry factor *g*_CPLE_, given by
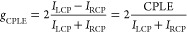
1where *I*_LCP_ and *I*_RCP_ represent the intensity of LCP and RCP emission, respectively and CPLE is defined as CPLE = *I*_LCP_*– I*_RCP_. An emission of LCP without its polarization counterpart gives *g*_CPLE_ = 2. In contrast, *g*_CPLE_ = −2 results from the emission of RCP alone. A mixture of LCP and RCP in a ratio of 1:1 causes *g*_CPLE_ = 0. The purer the circular polarization of CPLE, the larger the absolute value of *g*_CPLE_, which has a maximum absolute value of 2. One of the critical problems prohibiting the commercialization of CPLE devices is small *g*_CPLE_ value. The subwavelength chiral materials weakly interact with the excitation irradiation, so that the CPL-dominant CPLE usually has a |*g*_CPLE_| value on the order of 10^–5^–10^–3^.^18−26^ A dimensional amplification through supramolecular assemblies, chiral-ligand-directed bottom-up growth, and chiral template-directed assemblies leads to an obvious increase of |*g*_CPLE_| on the order of 10^–1^–10^0^,^5,13,27^ mainly ascribed to the CPS. Among diverse methods to produce the CPS, supramolecular assemblies of organic molecules into chiral nanospirals or nanohelices (NHs), which have a helical pitch (*P*) in the nanoscale, have been widely adopted to achieve a |*g*_CPLE_| value in a range of 10^–3^–10^0^.^28,29^ Supramolecular assemblies of lanthanides, cesium tetrakis(3-heptafluorobutylryl-(+)-camphorato) Eu(III) complexes, have a large *g*_CPLE_ value of 1.38, probably owing to the magnetic-dipole-allowed but electric-dipole-forbidden f–f transition, large pseudo-Stokes shift, and long luminescence lifetime.^30,31^ Inorganic NHs,^32,33^ fabricated by template-assisted electrosynthesis,^34^ focused electron/ion beam-induced deposition,^35,36^ colloidal nanohole lithography together with tilted angle deposition,^37^ and glancing angle deposition (GLAD),^32,38^ are composed of the nanoscale helicity^39^ and thus exhibit strong optical activities in terms of the differential extinction (including absorption, reflection, and scattering) of LCP and RCP incidences,^40,41^ with high environmental stability.^42^ It is indicated that inorganic luminophore NHs have strong, stable CPS-dominant CPLE with large |*g*_CPLE_| values. To the best of our knowledge, however, there is a lack of application of inorganic NHs to generate CPLE.

Here, we apply GLAD^43,44^ to fabricate semiconductor luminophore NHs^45^ made of cadmium selenide (CdSe) and ceria (CeO_2_) for the emission of CPLE in red and UV–blue spectral regions, respectively. Chiral ligands have been widely used to generate chiral CdSe quantum dots with an emission of the CPL-dominant CPLE in the visible region.^46^ Ceria has special physicochemical properties and will be potentially applied to biochemical catalysis,^47,48^ biosensing,^49^ antioxidation,^50^ antibacterial,^51,52^ and treatment of tumor and ischemia strokes.^53^ To the best of our knowledge, however, CPLE has yet been imposed on ceria to seriously limit its bioapplications in a wide range, even though ceria nanostructures have been fabricated to generate photoluminescence in the UV–blue region.^54−56^ In this work, CdSe and ceria NHs are deposited on supporting substrates by GLAD to emit CPLE. Facile control of substrate rotation during GLAD enables a flexible engineering of helical handedness and *P* in the nano/micrometer scales, resulting in a control of circular polarization state and a significant increase of the *g*_CPLE_ values as large as 3 orders of magnitude. CdSe and ceria NHs show maximum |*g*_CPLE_| values of 0.15 and 0.06, respectively. Numerical simulations using COMSOL Multiphysics were performed to calculate the CD and CPLE of the NHs uniformly assembled in a square lattice, showing that the simulation results are in good agreement with the experimental results. They reveal that the CPL and CPS make a complicated contribution to the CPLE. The GLAD technique provides a versatile NH-fabrication platform to generate and tune high-*g*_CPLE_ CPLE in a broad (UV–visible–NIR) spectral region.

## Results and Discussion

### CdSe NHs with Engineerable Optical Activities

**Figure 1:**
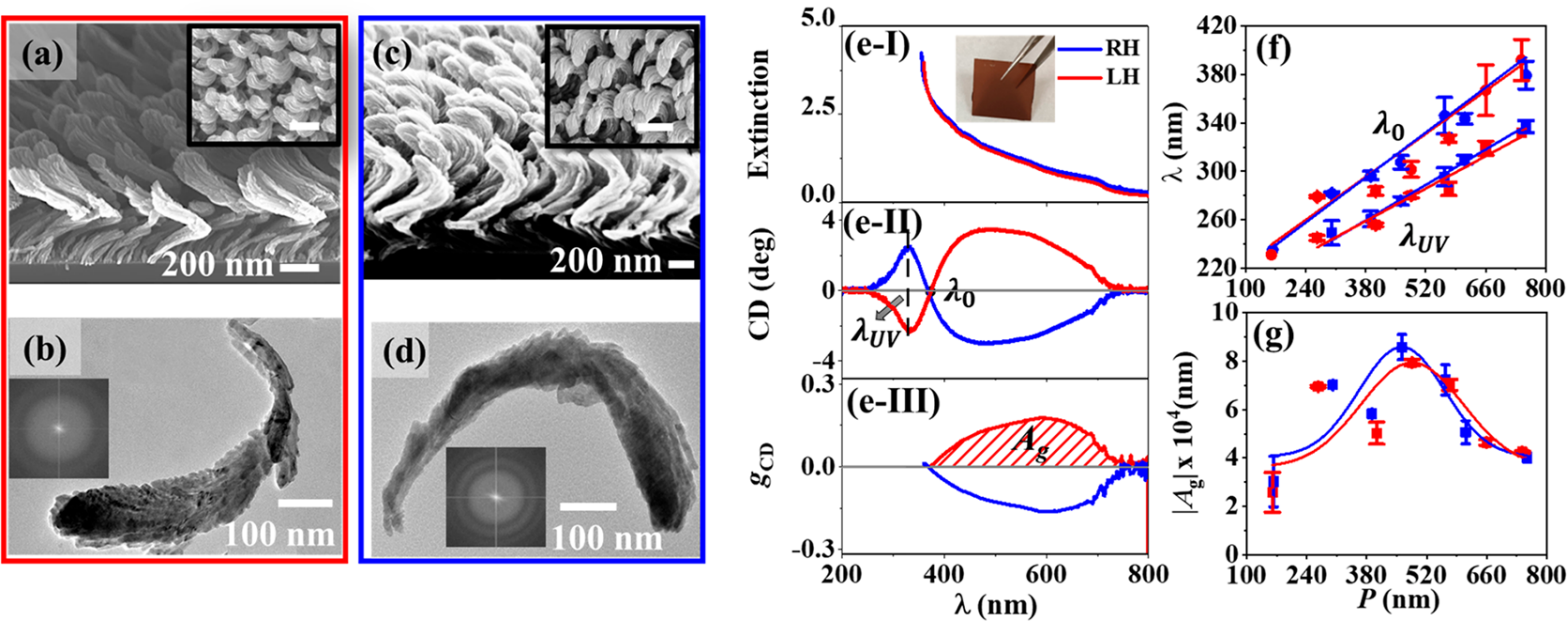
GLAD of one-turn CdSe NHs, having a helicity of (a, b) LH with *P* = 743 nm, and (c, d) RH with *P* = 754 nm. (a, c) Scanning electron microscopy (SEM) tilted images (insets, SEM top-down images; scale bar, 200 nm). (b, d) TEM images of individual CdSe NHs (insets, selected area electron diffraction (SAED) images). UV–visible–NIR spectra of the close-packed arrays of CdSe NHs: (e-I) extinction, (e-II) CD, and (e-III) *g*_CD_. (e-I) inset: a photograph of LH-CdSe NHs uniformly deposited on a sapphire with an area of 1.5 × 1.5 cm^2^. (f) Plots of λ_UV_ (the wavelength of the CD peak in the UV region, marked in e-II) and λ_0_ (the wavelength of the zero-crossing of the bisignated CD peaks, marked in e-II) versus *P*, which are linearly fitted. (g) Plots of the integrated area of the *g*_CD_ peak in the visible region (*A*_g_, marked with the shading in e-III) versus *P*, fitted with a Gaussian function. (f, g) Monitored parameters are shown as an average value with a standard deviation (represented with scale bars), with not less than three samples being monitored for the statistical analysis. (e–g) LH, red symbols; RH, blue symbols.

GLAD was performed to deposit a close-packed array of one-turn CdSe NHs with random assembly (Figure S1). Counterclockwise and clockwise substrate rotation in 360° during GLAD enabled the fabrication of left-handed (LH, Figure 1a) and right-handed (RH, Figure 1c) NHs in one turn, respectively.^57^ The rate of substrate rotation was controlled to engineer the *P* in a range of 160–760 nm (according to eq 5 (Materials and Methods) and Figure S1, I–VII). The NHs were evaluated to have an average atomic Cd:Se ratio of ∼2:3 (i.e., Cd_0.4_Se_0.6_), independent of the helicity (Figure S2). CdSe NHs are polycrystals (insets in Figure 1b,d, and Figure S3a–g) and composed of the grains that tend to shrink in size with an increase in *P* (Figure S3h). They appear to have a broadening profile along with their growth and possess rough and branching surfaces (Figure 1b,d). The close-packed array of CdSe NHs, vertically protruding on a supporting substrate in a random arrangement, shows UV–visible–NIR broad-band extinction (Figure 1e-I). Elongating *P* above 300 nm gradually reduces the optical transparency of the NH arrays deposited on sapphires (Figure S1d), where the extinction signals are out of the detection range in the UV region (Figure S1a). The optical activity of the CdSe NHs were characterized with circular dichroism (CD) in the UV–visible–NIR region, denoted as the differential extinction of incident LCP and RCP lights. The LH-CdSe NHs exhibit bisignated CD signals composed of a negative mode (CD < 0) in the UV region and a positive mode (CD > 0) in the UV–visible region (Figure 1e-II). The bisignated CD spectra flip around the zero-CD axis with a switch in the chirality from LH to RH, illustrating that the CdSe NHs possess intrinsic optical activity owing to their nanoscale helicity. The intrinsic optical activity tends to red shift with the elongation of *P* (Figure S1b). Both λ_UV_ (the peak wavelength of the CD mode in the UV region, Figure 1e-II) and λ_0_ (the zero-crossing wavelength of the bisignated CD peaks, Figure 1e-II) linearly shift with *P*. λ_UV_ and λ_0_ have a red-shift slopes of ∼0.20 and ∼0.26, respectively, which is nearly independent of the helical handedness (Figure 1f). It is not convenient to quantitatively study the *P*-induced red shift of another CD mode in the UV–visible region, because it appears to split into two peaks at some *P* value (Figure S1b, II–V). To quantitatively evaluate the optical activity of individual NHs in a close-packed array, the CD signals of a NH array were normalized by the extinction signals to evaluate the anisotropic *g* factor (or *g*_CD_), according to^58^
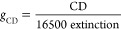
2where CD is the ellipticity (units: degree, or deg). Due to the saturation of the monitored extinction signals in the UV region when *P* > 300 nm, only the UV–visible CD mode could be evaluated in terms of *g*_CD_ with the elongation of *P* in the full range of 160–760 nm (Figure S1c). *g*_CD_ is evaluated to be as large as 0.4 in the visible region, 1 order of magnitude higher than the largest *g*_CD_ value for CdSe chiral nanostructures reported previously,^59^ to the best of our knowledge (Table S1). The integrated area of the UV–visible *g*_CD_ peak (*A*_g_, Figure 1e-III) was calculated as a function of *P*, approximately showing a volcano profile that reaches the maximum optical activity at a *P* ≈ 480 nm (Figure 1g).

**Figure 2:**
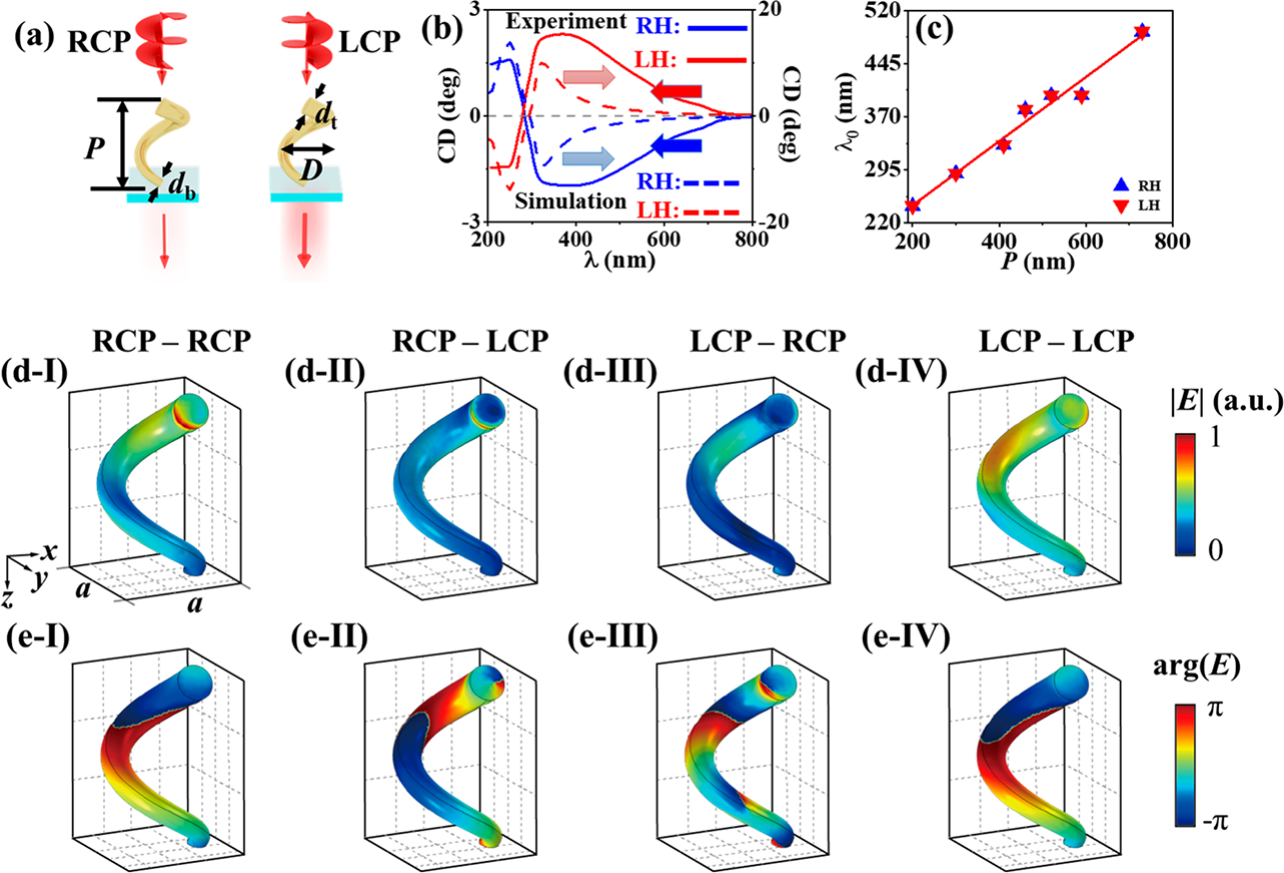
Numerical simulations of CD in a periodic assembly of the one-turn CdSe NHs. (a) Schematics for simulating the CD of an LH-CdSe NH excited with RCP and LCP incidences, while the transmitted light is simulated. A helical structure is presented with a helical pitch (*P*), wire diameter on the top (*d*_t_) and at the bottom (*d*_b_), and coil diameter (*D*). (b) Comparison of the simulated CD spectra (dashed lines) and the experimental results (solid lines) in the UV–visible–NIR region, for the LH- and RH-CdSe NHs with *P* ≈ 300 nm. (c) Plot of the simulated λ_0_ versus *P*, with a linear fitting. (b, c) LH, red symbols; RH, blue symbols. In the simulation, a square lattice with a period of *a* (d-I) was constructed to accommodate individual NHs. The distribution of electric field (**E**, at a wavelength of 320 nm) at the helical surfaces is simulated in terms of (d) the amplitude and (e) phase: (I) RCP-RCP (incident and transmitted light both with the RCP state); (II) RCP-LCP (incident and transmitted light with the RCP and LCP state, respectively); (III) LCP-RCP (incident and transmitted light with the LCP and RCP states, respectively); (IV) LCP-LCP (incident and transmitted light both with the LCP state).

With an incidence of RCP or LCP irradiation from the top of individual CdSe NHs vertically protruding on a sapphire substrate, the transmission light was simulated according to the experimental CD measurement (Figure 2a, and Materials and Methods, Numerical Simulation). One NH with the broadening profile was modeled with the helicity composed of *P*, coil diameter *D*, and wire diameter gradually increasing from the bottom (*d*_b_) to the top (*d*_t_) along with the helical growth. The random assembly of the NHs was approximately modeled with a periodic assembly in a square lattice (with a period of *a*, Figure 2d-I). All of these modeling parameters are summarized in Table S2, according to the structural characterization with SEM. It is very challenging to model CdSe NHs made of a rough, branching surface, and thus smooth surfaces were constructed in the modeling.

The simulated CD spectra qualitatively have good agreement with the experimental results (Figure 2b and Figure S4a versus Figure S4b). The simulation results show the bisignated CD features that have a red shift with the elongation of *P*. Some deviations from the experimental results were observed. The simulations reveal that the CD peak in the UV region tends to split into two peaks when *P* > 400 nm and that in the UV–visible region does not. In contrast, it was experimentally monitored that the peak splitting occurred in the UV–visible region in the *P* range of 400–660 nm but did not occur in the UV region. The simulated CD intensities are stronger than the experimental results, and the red-shift slope of λ_0_, which was simulated to be 0.45 (Figure 2c), is larger than the experimental result (∼0.26, Figure 1f). The *A*_g_ values of the UV–visible *g*_CD_ peak were simulated to linearly increase with the elongation of *P* in the range of 200–520 nm, reach a plateau in the *P* range of 520–590 nm, and then continuously increase at *P* > 590 nm (Figure S5). However, the experimental results showed that *A*_g_ had a volcano profile with *P* (Figure 1g). These deviations can probably be ascribed to those in the structural modeling. The CdSe NHs were modeled with the smooth surfaces in a periodic assembly, so that the complicated optical scattering from the rough, branching surfaces and in the random assembly could not be simulated.

The amplitude and phase distributions of the near-field polarization, which contribute to the far-field transmission from each local point of the LH-CdSe NH (i.e., with *P* = 300 nm), were calculated (Figure 2d,e, respectively). The amplitudes of the RCP-LCP and LCP-RCP components (Figure 2d-II,d-III) are much weaker than those of the RCP-RCP and LCP-LCP components (Figure 2d-I,d-IV). The phase distribution shows a significant difference for the incident and transmitted light with polarization combinations (Figure 2e), and the intensity of the far-field transmitted light depends on where the constructive or destructive interference conditions are satisfied. It should be noted that the incident light experiences complicated scattering processes in the randomly close-packed arrays, leading to the complicated amplitude and phase distributions. Therefore, it is challenging to explicitly correlate the near-field information with the measured CD values. It further illustrates that the differential extinction, including not only the absorption by the NHs but the scattering in the random assembly, accounts for the measured CD signals.

### Strong, Tunable CPLE of CdSe NHs

**Figure 3:**
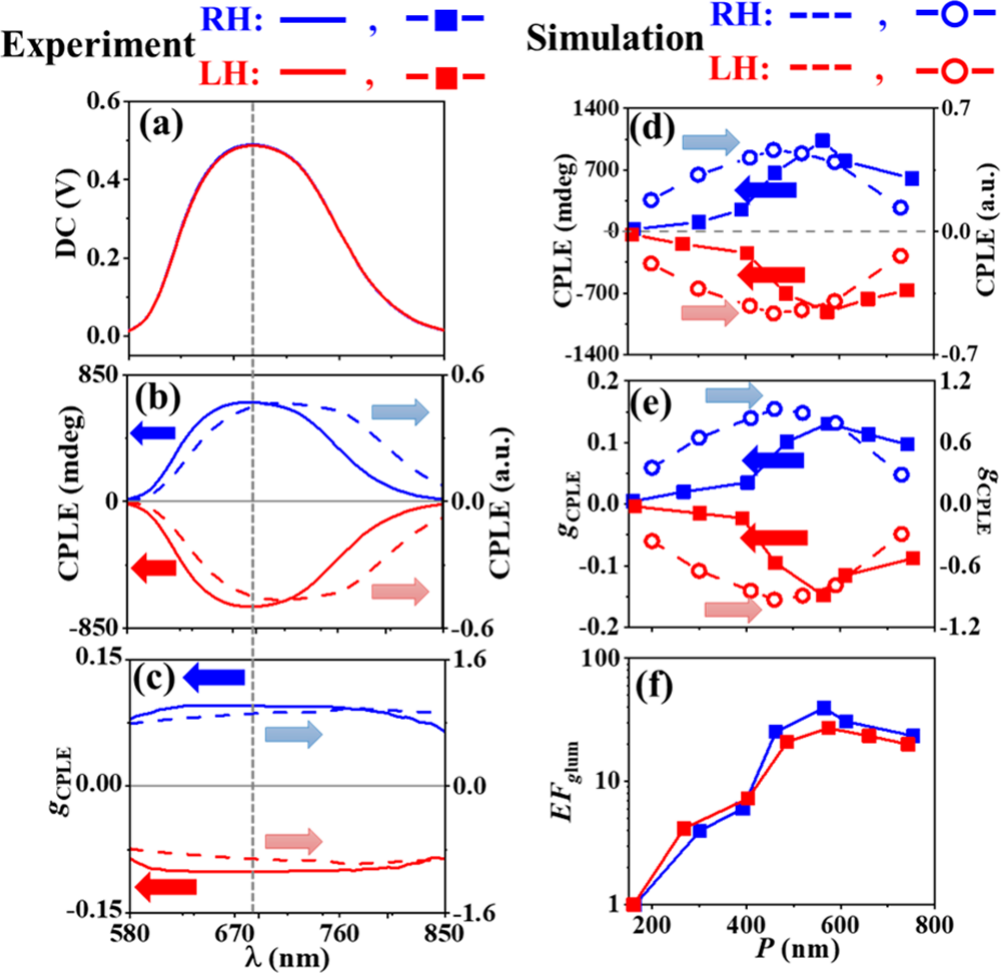
CPLE of one-turn LH (with *P* = 486 nm) and RH (with *P* = 462 nm) CdSe NHs, characterized with (a) photoluminescence, (b) CPLE, and (c) *g*_CPLE_ spectra. (b, c) CPLE and *g*_CPLE_ spectra simulated with *P* = 460 nm for both LH- and RH-CdSe NHs. Plots of (d) CPLE, (e) *g*_CPLE_, and (f) EF_*g*_CPLE__ measured at a wavelength of ∼688 nm (marked with a gray dashed line in (a–c) and Figure S6) versus *P* in a range of 160–760 nm. (a–f) LH, red symbols; RH, blue symbols. Experimental results: (a–c) solid lines and (d–f) solid lines with solid squares. Simulation results: (b, c) dashed lines and (d, e) dashed lines with hollow circles.

Under a 532 nm excitation, the CdSe NHs emit red photoluminescence at a wavelength of ∼688 nm (Figure 3a). The photoluminescence profile appears to be independent of the helical chirality (LH and RH), and the red photoluminescence barely shifts with the elongation of *P* (Figure S6a). The CdSe NHs are luminophores and show strong optical activity with respect to the differential extinction (including absorption, reflection, and scattering) of LCP and RCP irradiations, so they could be CPLE active owing to the CPL and CPS. The CdSe NHs show red CPLE on resonance with their photoluminescence; according to eq 1, RCP and LCP lights are preferentially emitted from the LH- and RH-CdSe NHs, respectively (Figure 3b). Switching the chirality from LH to RH causes the CPLE spectrum to flip around the zero-CPLE axis (Figure S6b). The CPLE values at the peak wavelength of ∼688 nm also approximately show a volcano-like profile with the elongation of *P*, reaching the maximum value of 1036 and −914 mdeg for the RH (at *P* = 564 nm) and LH (at *P* = 574 nm) CdSe NHs, respectively (Figure 3d). According to eq 1, the *g*_CPLE_ spectra were calculated as a function of *P* (Figure 3e and Figure S6c). *g*_CPLE_ at ∼688 nm shows a volcano profile with an increase of *P*, and the |*g*_CPLE_| values reach the maximum value of ∼0.15 at a *P* ≈ 570 nm (Figure 3e). With a comparison to the smallest *g*_CPLE_ value measured at a *P* ≈ 160 nm (or *g*_CPLE_(*P* ≈ 160 nm), an enhancement factor (EF_*g*_CPLE__) was calculated by
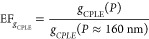
3where *g*_CPLE_(*P*) represents the *g*_CPLE_ value for a given *P*, measured at the wavelength of ∼688 nm. EF_*g*_CPLE__ reaches a maximum value of ∼40 at *P* ≈ 570 nm, that is, the elongation of *P* from ∼160 to ∼570 nm results in a 40-fold amplification of *g*_CPLE_ (Figure 3f). The CdSe NHs have a moderate *g*_CPLE_ value, compared to those reported for the CdSe chiral nanostructures (Table S3). For those to achieve *g*_CPLE_ larger than this work, multiple fabrication processes were generally performed, including the synthesis of luminophore quantum dots, fabrication of chiral templates, and chiral template-assisted assembly of the quantum dots. In this work, the one-step GLAD was applied to generate the CdSe NHs, which will be facilely adapted to mass production of CPLE devices for advanced optic applications.

It has been widely studied that the optic dissymmetry *g* factor of CPL (i.e., *g*_lum_) emitted from the subwavelength chiral molecules is proportional to *g*_CD_.^60^ However, the *g*_CPLE_ values measured at the peak wavelength of ∼688 nm show a variation with *P* obviously different from the *g*_CD_ values measured at the CPLE-excitation wavelength of 532 nm (Figure S7). It is illustrated that the CPS plays a significant role in the CPLE of the CdSe NHs with a *P* value comparable to the excitation wavelength through Mie scattering of the incident light in a chiral, inelastic manner and that of the CPL emitted from the CdSe NHs in a chiral, elastic/inelastic way.

**Figure 4:**
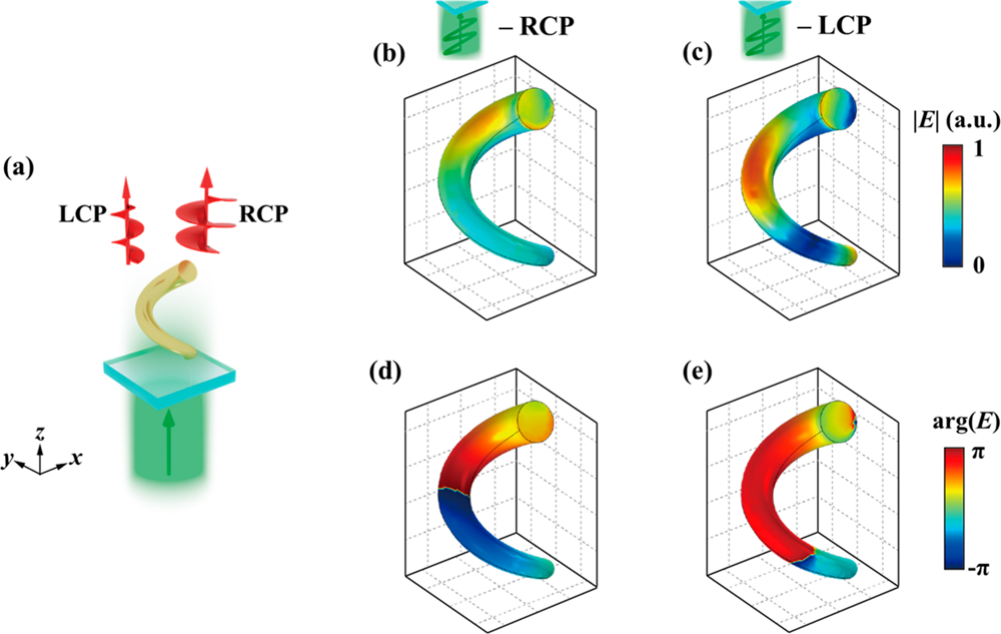
Numerical simulation of CPLE of the one-turn LH-CdSe NHs having *P* = 460 nm. (a) Schematic of simulating the CPLE of a LH-CdSe NH excited with a nonpolarized light at a wavelength of 532 nm, while the emission lights with the LCP and RCP states are simulated. The distribution of electric field (**E**, at the wavelength of 690 nm) at the helical surfaces simulated in terms of (b, c) the amplitude and (d, e) phase. In the simulation, the nonpolarized incidence is composed of eight linearly polarized lights with a polarization angle interval of 22.5°. (b–e) The incident light is linearly polarized along the *x* axis, and the emission light is in the (b, d) RCP and (c, e) LCP state.

To study the CPLE mechanisms, numerical simulations were performed with an incidence of monochromatic nonpolarized light from the bottom of a CdSe NH to simulate the emission of the LCP and RCP lights (Figure 4a), according to the experimental measurement of CPLE. The nonpolarized light was simulated to be composed of eight linearly polarized lights with a polarization angle interval of 22.5°. The simulated results (Figure S8) are in good agreement with the experimental measurements, with respect to the spectral profiles (Figure 3b,c, respectively) and the volcano profiles of CPLE and *g*_CPLE_ versus *P* (Figure 3d,e, respectively). Some deviations were also observed: the simulated CPLE spectra tend to have a red shift with the experimental results, the volcano peaks were simulated to be located at a shorter *P* compared to the experimental measurements, and the simulated *g*_CPLE_ values are generally larger than the experimental values in the whole *P* range. These deviations can probably be ascribed to those in the helical modeling.

To better understand the far-field intensity of the CPLE, we also calculated the near-field distribution of the electric fields of LH-CdSe NH (*P* = 460 nm). As an example, linearly polarized (along the *x* axis) pump light at a wavelength of 532 nm was used as the excitation light. In the simulation model, the propagation effect of both the pump light and photoluminescence are taken into account. The calculated amplitude (Figure 4b,c) and phase (Figure 4d,e) distributions of the RCP and LCP emission light at a wavelength of 690 nm are shown in Figure 4b–e. At each local point of the NH, we can assume that there is a dipole emitter which has spatially variant amplitude and phase. The emitted light from each point will pass through the NH arrays and be reflected or scattered. Therefore, it is very difficult to simply tell which circularly polarized component is stronger until calculating the integrated local fields, even though the local field distributions of the photoluminescence light can be calculated. The numerical simulation further verifies that both the CPL and CPS contribute to the observed CPLE.

### Chiroptically Active Ceria NHs with Controllable CPLE

**Figure 5:**
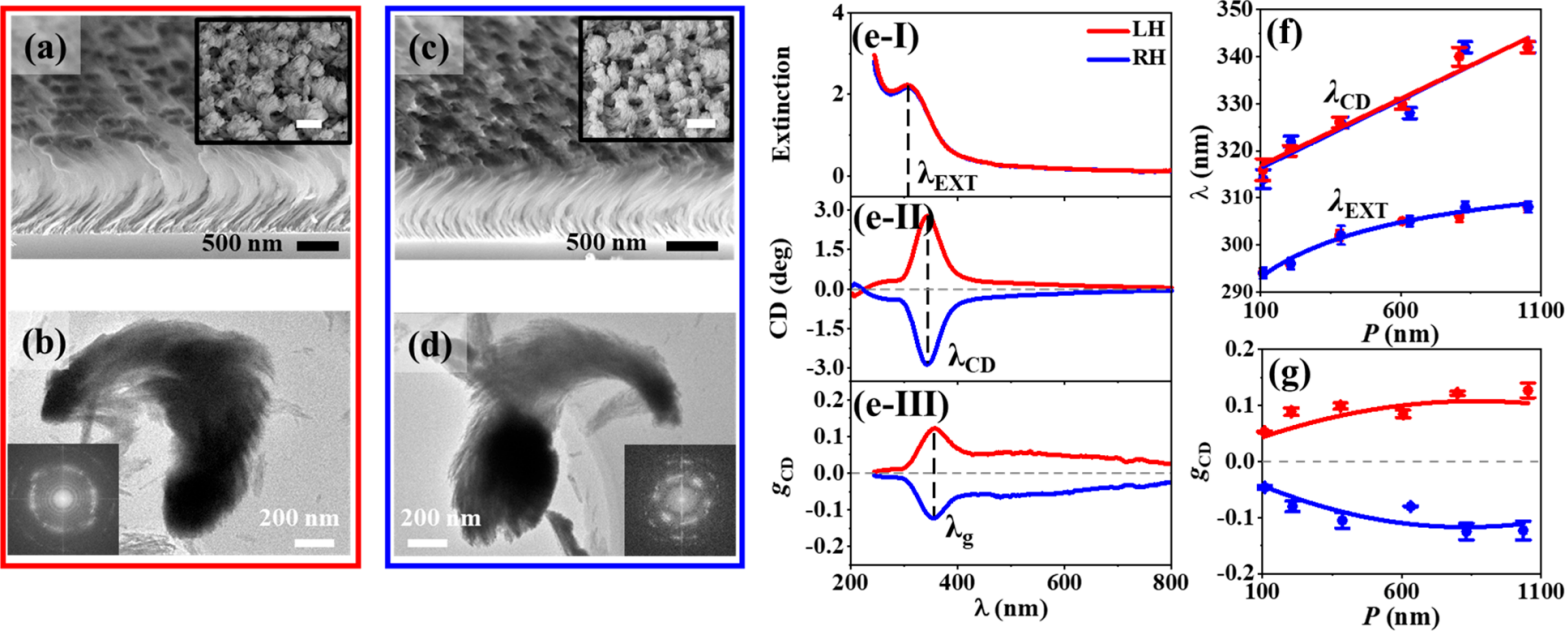
GLAD of one-turn ceria NHs: (a, b) LH with a *P* of 1053 nm, (c, d) RH with a *P* of 1036 nm. (a, c) SEM tilted images (insets, SEM top-down images; scale bar, 500 nm). (b, d) TEM images of individual ceria NHs (insets: SAED images). UV–visible–NIR spectra of the close-packed arrays of ceria NHs: (e-I) extinction, (e-II) CD, and (e-III) *g*_CD_. (f) Plots of λ_EXT_ and λ_CD_ (marked in (e-I) and (e-II), respectively) versus *P*. The plot of λ_CD_ is linearly fitted, and the plot of λ_EXT_ is fitted with an exponential function. (g) Plots of *g*_CD_ measured at λ_g_ (marked in (e-III)) versus *P*, fitted with a parabolic function. (f, g) Monitored parameters shown in an average value and a standard deviation (shown in scale bars), with not less than three samples being monitored for the statistical analysis. (e–g) LH, red symbols; RH, blue symbols.

It is a practical demand to produce CPLE emitted in a controlled spectral region. For instance, UV–blue CPLE can be generated from the ceria NHs. GLAD was performed to fabricate a close-packed array of one-turn ceria NHs vertically protruding on a supporting substrate in a random assembly (Figure 5a,c), and *P* was tuned in a wide range of 100–1100 nm. The ceria NHs are polycrystalline and have fluorite ceria structures with dominant crystal orientation direction along ⟨111⟩ (Figure S9a–f and insets in Figure 5b,d), and their grain size tends to increase with the elongation of *P* (Figure S9g). Monitored with X-ray photoelectron spectroscopy (XPS), the stoichiometric *y* value of oxygen atoms in ceria (CeO_*y*_) was evaluated to increase from 1.8 to 1.9 with the elongation of *P* (Figure S10), indicating the existence of oxygen vacancies in the ceria NHs. Analogous to the CdSe NHs, the ceria NHs have rough, branching surfaces with the broadening profile (Figure 5b,d). They show extinction (Figure 5e-I) and CD (Figure 5e-II) signals mainly in the UV–blue region (Figure S11a,b), where switching the helicity from LH to RH generally caused the CD spectra to flip around the zero-CD axis. Therefore, the ceria NHs intrinsically are optically active due to their nanohelicity. In the broad *P* range, the extinction peak wavelength (λ_EXT_, Figure 5e-I) is generally shorter than the CD peak wavelength (λ_CD_, Figure 5e-II); the elongation of *P* results in a red shift of λ_EXT_ from 294 to 308 nm and that of λ_CD_ from 310 to 345 nm (Figure S11). λ_CD_ tends to have a linear red shift with the elongation of *P* (fitted with a red-shift slope of 0.03), and λ_EXT_ appears to deviate from the linear variation (Figure 5f). The |*g*_CD_| values measured at the peak wavelength (λ_g_, Figure 4e-III) tends to increase from 0.05 at *P* ≈ 100 nm to 0.13 at *P* ≈ 800 nm, followed by reaching a plateau of 0.13 in the *P* range of 800–1100 nm (Figure 5g). The variation of |*g*_CD_| and the red shift of λ_EXT_ and λ_CD_ induced by the elongation of *P* appear to be independent of the helical handedness.

**Figure 6:**
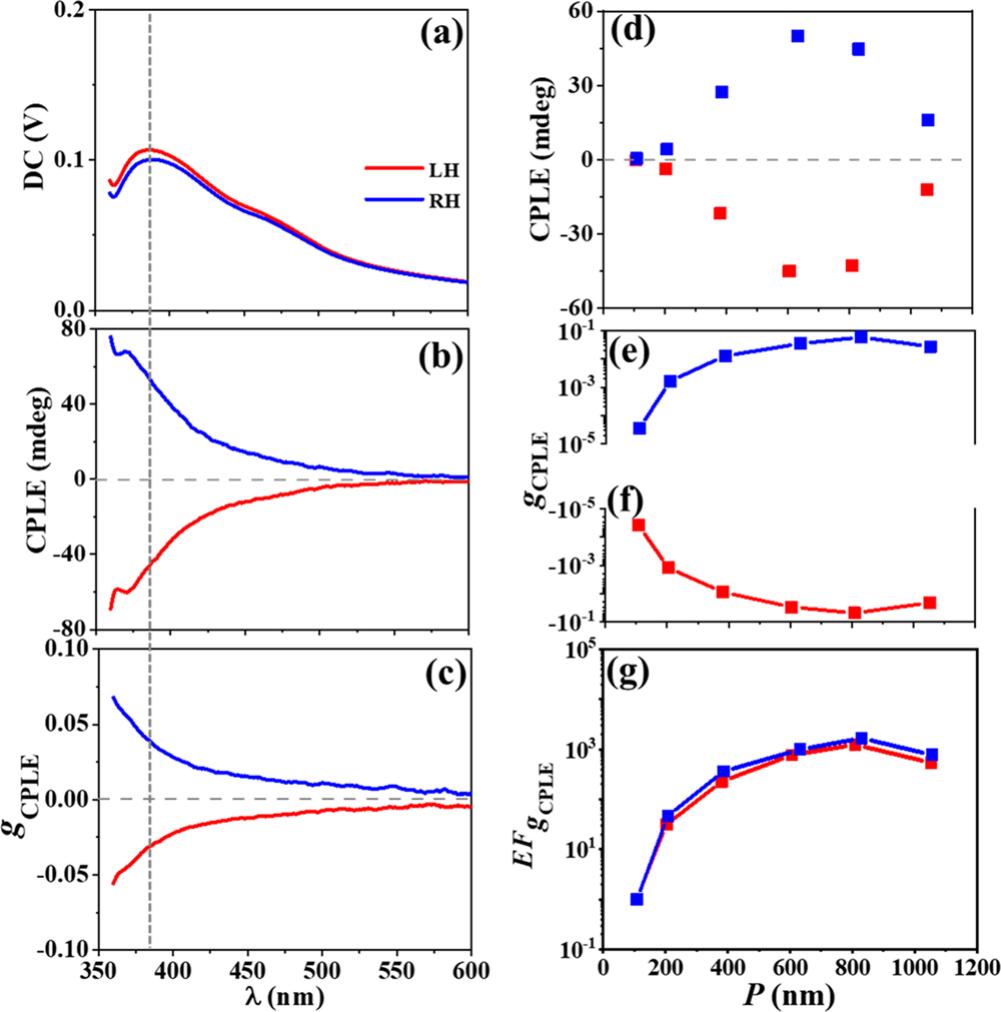
CPLE of one-turn LH (with *P* = 605 nm) and RH (with *P* = 632 nm) ceria NHs, characterized with (a) photoluminescence, (b) CPLE, and (c) *g*_CPLE_ spectra. Plots of (d) CPLE, (e, f) *g*_CPLE_, and (g) EF_*g*_CPLE__ measured at a wavelength of ∼390 nm (marked with a black dashed line in (a–c) and Figure S12) versus *P* varying in a range of 100–1100 nm. (a–g) LH, red symbols; RH, blue symbols.

Under the 320 nm excitation, the ceria NHs emit a UV–visible broad-band photoluminescence with a peak at ∼390 nm, nearly independent of the helical handedness (Figure 6a and Figure S12a). The ceria NHs emit CPLE mainly in the UV–blue region, which quenches quickly in the visible region (Figure 6b and Figure S12b). Analogous to the CdSe NHs, the LH- and RH-ceria NHs preferentially emit RCP and LCP light, respectively. Both the CPLE intensity and *g*_CPLE_ (Figure 6c) monitored at ∼390 nm shows a volcano profile with the elongation of *P* (Figure 6d and Figure 6e,f respectively). For the RH-ceria NHs, the *g*_CPLE_ value reaches the maximum of 0.06 at *P* = 830 nm, which has a EF_*g*_CPLE__ value of 1.7 × 10^3^ (Figure 6g). The *g*_CPLE_ value of the LH-ceria NHs reaches the maximum of −0.05 at *P* = 809 nm, which has a EF_*g*_CPLE__ value of 1.3 × 10^3^. The enhancement factor EF_*g*_CPLE__ for the ceria NHs is calculated by
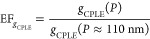
4where *g*_CPLE_(*P*) and *g*_CPLE_(*P* ≈ 110 nm) represent the *g*_CPLE_ values for a given *P* and *P* ≈ 110 nm, respectively. The RH- and LH-ceria NHs have a *g*_CPLE_ value of 3.5 × 10^–5^ at *P* = 109 nm and 3.9 × 10^–5^ at *P* = 108 nm, respectively. Therefore, the CPLE of ceria NHs can be facilely enhanced in 3 orders of magnitude with respect to *g*_CPLE_, through elongating *P* from ∼110 to ∼800 nm. Analogous to the CdSe NHs, the variation of *g*_CPLE_ as a function of *P* obviously differs from that of *g*_CD_ measured at the CPLE excitation wavelength of 320 nm (Figure S13), illustrating that the CPLE can be attributed to not only the CPL but also the CPS.

Different from the CdSe NHs, the ceria NHs appear to consist of numerous nanowhiskers growing from their helical cores (Figure 5b,d). The modeling of the ceria NHs with smooth helical surfaces, analogous to that of the CdSe NHs, made the simulation results of CD and CPLE significantly deviate from the experimental measurements. This further illuminates that the CPS, stemming from the nanowhiskers, plays an essential role in the CD and CPLE of the ceria NHs.

## Conclusions

GLAD enables the one-step fabrication of diverse inorganic NHs to emit CPLE with material-determined color: for example, the red and UV–blue CPLE emitted from the CdSe and ceria NHs, respectively. The circular polarization for CD and CPLE is simply changed by rotating a substrate clockwise or counterclockwise during the GLAD of inorganic NHs on the substrate. The CPLE of luminophore inorganic NHs, which have nanoscale helicity comparable to the excitation wavelength, is ascribed to the Mie scattering of the incident light in a chiral, inelastic manner and that of the CPL emitted from the NHs in a chiral, elastic/inelastic way. The *g*_CPLE_ values sensitively depend on *P* that is controlled with the rate of substrate rotation, showing a volcano profile with the elongation of *P*. The CdSe NHs show a *g*_CD_ value of 0.4, the highest optical activity compared to other chiral CdSe nanostructures previously reported, and have a moderate *g*_CPLE_ value of 0.15 at *P* ≈ 570 nm, with a 40-fold amplification of *g*_CPLE_ compared to that at *P* ≈ 160 nm. The ceria NHs have a *g*_CD_ value of 0.13, and show a *g*_CPLE_ value of ∼0.06 at *P* ≈ 830 nm, with a 10^3^-fold amplification compared to that at *P* ≈ 110 nm. The GLAD-based nanofabrication platform is devised to produce inorganic NHs with high optical activity (*g*_CD_ on an order of 10^–1^) for the emission of CPLE having high *g*_CPLE_ values in a range of 10^–2^–10^–1^. To understand the measured results, we developed a simulation model using COMSOL Multiphysics, in which the propagation effect of the pump wave was taken into account. Therefore, the photoluminescence behavior of the randomly packed NHs can be accurately calculated by assuming that they are uniformly assembled in a square lattice. Due to the complicated propagation effect of light in the three-dimensional helical structures (ascribed to the CPL and CPS), it is difficult to intuitively understand the relationship of the near-field distributions of light and their far-field intensities. However, the theoretical model developed in this work will be useful to further optimize the optical performance of the NHs.

The GLAD technique has been adapted to the fabrication of a wide range of inorganic semiconductor nanostructures with diverse band gaps^61^ and has been demonstrated for large-area, uniform, repeatable fabrication.^62^ The circular polarization of CPLE can be simply changed by rotating a substrate clockwise and counterclockwise, the purity of circular polarization (or *g*_CPLE_) can be significantly amplified in 3 orders of magnitude by adjusting the substrate rotation rate to tune *P*, and the CPLE color can be flexibly tailed with semiconductor materials. Therefore, this work devises a versatile NH-fabrication platform that will potentially promote the mass production of CPLE devices with promising applications in the fields of 3D display, information storage, anticounterfeiting, bioimaging, and biometry.

## Materials and Methods

### GLAD

In a custom-built physical vapor deposition chamber (JunSun Tech Co. Ltd., Taiwan), GLAD was performed at a deposition angle (α) of 86° (with respect to the normal direction of a substrate) and in a high vacuum of 10^–7^–10^–6^ Torr. CdSe (99.99%, Fuzhou Innovation optoelectronic Technology Co., Ltd.) and CeO_2_ (99.99%, Fuzhou Innovation optoelectronic Technology Co., Ltd.) were applied with electron-beam evaporation to condense on silicon wafers (Semiconductor Wafer, Inc.) and sapphires (MTL, Hong Kong) in an area of 1.5 × 1.5 cm^2^. The deposition rate (*R*_d_) was monitored by a quartz crystal microbalance to be 4 Å/s for CdSe and 3 Å/s for CeO_2_, at an electron-beam accelerating voltage of 8.0 kV and emission currents of 4–8 mA for CdSe and 25–30 mA for CeO_2_. During GLAD, the substrate temperature was controlled at ∼0 °C using a water-cooling system. The LH and RH NHs were sculpted by rotating substrates in counterclockwise and clockwise, respectively. *P* (in units of nm per revolution) can be engineered by
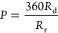
5where *R*_r_ is the substrate rotation rate (in units of degrees per second). *R*_d_ was calibrated as 1.5 Å/s for CdSe and 1.7 Å/s for CeO_2_, with respect to an α value of 86°. The helical *P* was experimentally evaluated with
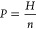
6where *H* is the helical height and *n* is the number of helical turns (equal to the number of substrate rotations during GLAD). In this work, the NHs were composed of one helical turn (or *n* = 1) so that *H* = *P*. *H* was measured with SEM (Oxford, LEO 1530) cross-sectional images, whereby multiple positions of a sample were monitored to obtain an average *H* (or *P*) value. The CdSe and ceria NHs had *P* values in the ranges of 160–760 and 100–1100 nm, respectively.

### Optical Characterization

Bio-Logic CD (MOS 500) and DSM 1000 CD (Olis Inc.) were used to monitor the UV–visible extinction and CD spectra of the NHs deposited on sapphire, respectively, with an incidence along the normal direction of the sapphires. CPLE spectra of the close-packed NH arrays deposited on sapphires were monitored with a JASCO CPL-300 Spectro under ambient conditions, at a scanning speed of 200 nm/min with the “Continuous” mode. Both the CD and CPLE spectra were monitored with transmission mode. The CdSe NHs were excited with a nonpolarized 532 nm light along the normal direction of the sample, and a 650 nm optical filter was placed behind the measured sample. A nonpolarized 320 nm irradiation was applied to the ceria NHs, and a 360 nm optical filter was placed behind the sample. The “slit” mode was applied to monitor CPLE spectra, with an Ex slit width of 3000 μm and an Em slit width of 3500 μm. To eliminate the disturbance of linear birefringence and linear dichroism to CD and CPLE due to the anisotropic growth orientation of the protruding NHs, the CD and CPLE spectra were monitored using the following procedure. For one sample, four spectra in the UV–visible–NIR region were subsequently recorded. After a spectrum was monitored, the sample was manually rotated at an angle of 90° around its normal axis before measuring the next spectrum. Then, the four spectra were algebraically averaged to obtain a spectrum of the sample to eliminate the linearly anisotropic effects. It was monitored that the rotation of a sample (e.g., the ceria NHs) had a negligible effect on the CD and CPLE spectra (Figure S14). This illustrates that the close-packed NH arrays possess optical activities barely disturbed by the linearly anisotropic effects.

### Nanomaterial Characterization

The as-deposited samples were mechanically split, leaving the freshly exposed surfaces for the characterization by SEM (Oxford, LEO 1530). The inorganic NHs were scratched off the substrates and dispersed well in ethanol via ultrasonication for 15 min. Several drops of the mixture were applied to a transmission electron microscope (TEM) grid with lacey carbon film (Electron Microscopy Sciences). The grid was dried under ambient conditions and then characterized by TEM with a SAED instrument (Tecnai G2 20 STWIN). With no postdeposition treatment, the samples were characterized by X-ray diffraction (XRD, Bruker, nonmonochromated Cu Kα X-rays with a wavelength of 0.15418 nm, Advance D8 multipurpose X-ray diffractometer) and XPS (performed in an ultrahigh-vacuum surface analysis system equipped with an ULVAC PHI 5000 VersaProbe III spectrometer, monochromatic Al Kα radiation of 1486.6 eV).

### Numerical Simulation

The COMSOL Multiphysics software was used for the numerical simulation of CD and CPLE. The simulations were simplified by an assumption that the inorganic NHs in the randomly distributed close-packed arrays are periodically arranged in a square lattice with a period of *a*, using the refractive index of CdSe previously reported.^63^ The periodic boundary conditions were set in the *x* and *y* directions, and the perfectly matched layers were set in the *z* direction. The helical nanostructures were defined by the parameters summarized in Table S2, which were measured from the SEM images. In the COMSOL Multiphysics software, the electric field vector of the LCP is defined as *E*_0_(*ê*_*x*_ + *iê*_*y*_)/√2 when power flow is along the positive *z* direction, where *ê*_*x*_ and *ê*_*y*_ are the unit vectors along the *x* and *y* directions, respectively.

In the simulation of CD, the LCP or RCP light is incident from the top of the vertically protruding NHs (Figure 2a). The electric fields of the incident light in the NHs and the output plane were recorded, and then the LCP and RCP components were calculated. Thus, CD measured in a degree of ellipticity θ can be written as
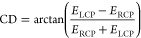
7where *E*_LCP_ and *E*_RCP_ are the amplitudes of the electric fields under LCP and RCP incidence, respectively.

In the simulation of CPLE (Figure 4a), the electric field was obtained first by an incident monochromatic nonpolarized light consisting of eight linearly polarized lights with a polarization angle interval of 22.5° on the NHs. Then the polarization of the electric dipole in the NHs was deduced from **P** = ε_0_χ**E**, where χ is the electric susceptibility and is set as unity for simplification. Afterward, the LCP and RCP components in the emission spectrum were retrieved by making the electric dipole the source of photoluminescence. Accordingly, CPLE and *g*_CPLE_ were calculated by
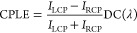
8
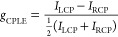
9where *I*_LCP_ and *I*_RCP_ are the intensities of emitted LCP and RCP light, respectively, and DC(λ) is the normalized experimental DC (or photoluminescence) spectrum which reflects the contribution of photoluminescence from unstructured materials.

## Supporting Information Available

Characterizations of nanostructures, optical activity, compositions, crystallinities, CPLE of CdSe and ceria NHs as a function of *P*, and numerical simulations (PDF)
