# Supplementary material for: Significant Enhancement of Circular Polarization in Light Emission through Controlling Helical Pitches of Semiconductor Nanohelices
Source: ACS Nano. 2023 Oct 5;17(20):20611–20. doi: 10.1021/acsnano.3c07663 (PMC10604094; doi:10.1021/acsnano.3c07663)
Supplement: Supplementary file 1 — nn3c07663_si_001.pdf [file nn3c07663_si_001.pdf]

Supporting Information

Significant Enhancement of Circular Polarization in Light Emission through Controlling Helical Pitches of Semiconductor Nanohelices

Ziyue Ni,[#,†] Ping Qin,[#,†] Hongshuai Liu,[#,†] Jiafei Chen,[#,♣,♦] Siyuan Cai,[‡] Wenying Tang,[¶] Hui Xiao,[‡] Chen Wang,[¶] Geping Qu,[‡,§] Chao Lin,[ǁ] Zhiyong Fan,[¶] Zong-Xiang Xu,[‡] Guixin Li[♣] and Zhifeng Huang[,⊥]*

[†]Department of Physics, Hong Kong Baptist University (HKBU), Kowloon Tong, Kowloon, Hong Kong SAR, China

[♣]Department of Materials Science and Engineering, Southern University of Science and Technology (SUSTech), Shenzhen, Guangdong 518000, China

[♦]School of Science, Harbin Institute of Technology, Shenzhen 518055, China

[‡]Department of Chemistry, SUSTech, Shenzhen, Guangdong 518000, China

[¶]Department of Electronic and Computer Engineering, The Hong Kong University of Science and Technology, Clear Water Bay, Kowloon, Hong Kong SAR, China

[§]School of Chemistry and Chemical Engineering, Harbin Institute of Technology, Harbin 150001, China

[ǁ]Department of Physics, The Chinese University of Hong Kong (CUHK), Shatin, New Territories, Hong Kong SAR, China

[⊥]Department of Chemistry, CUHK, Shatin, New Territories, Hong Kong SAR, China

zfhuang@cuhk.edu.hk

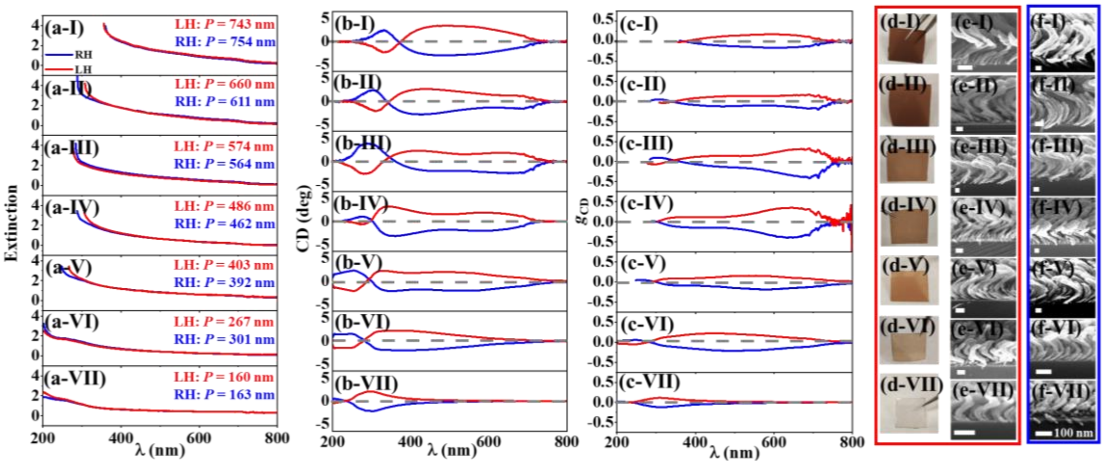

Figure S1. GLAD of one-turn CdSe NHs as a function of *P*: (I) 743 and 754 nm, (II) 660 and 611 nm, (III) 574 and 564 nm, (IV) 486 and 462 nm, (V) 403 and 392 nm, (VI) 267 and 301 nm, (VII) 160 and 163 nm, for LH and RH, respectively. UV–visible-NIR spectra of (a) extinction, (b) CD and (c) g_{CD} (LH: red lines; RH: blue lines). (d) Photographs of LH-CdSe NHs deposited on sapphires with an area of 1.5×1.5 cm^2. SEM tilted images of (e) LH- and (f) RH-CdSe NHs (scale bars: 100 nm).

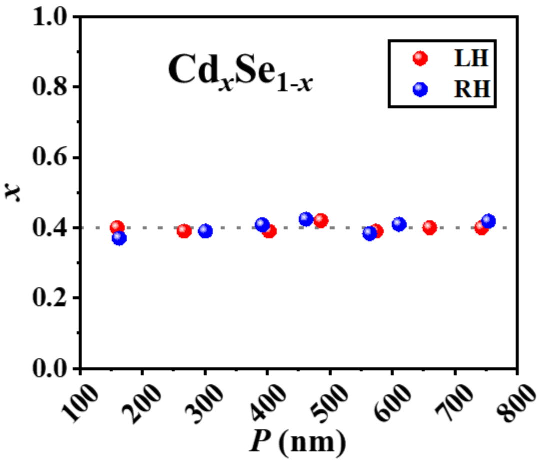

Figure S2. Plot of the stoichiometric *x* in the Cd_xSe_{1-x} NHs versus *P*, evaluated by energy-dispersive X-ray spectroscopy (EDS) installed in SEM. LH: red spheres; RH: blue spheres.

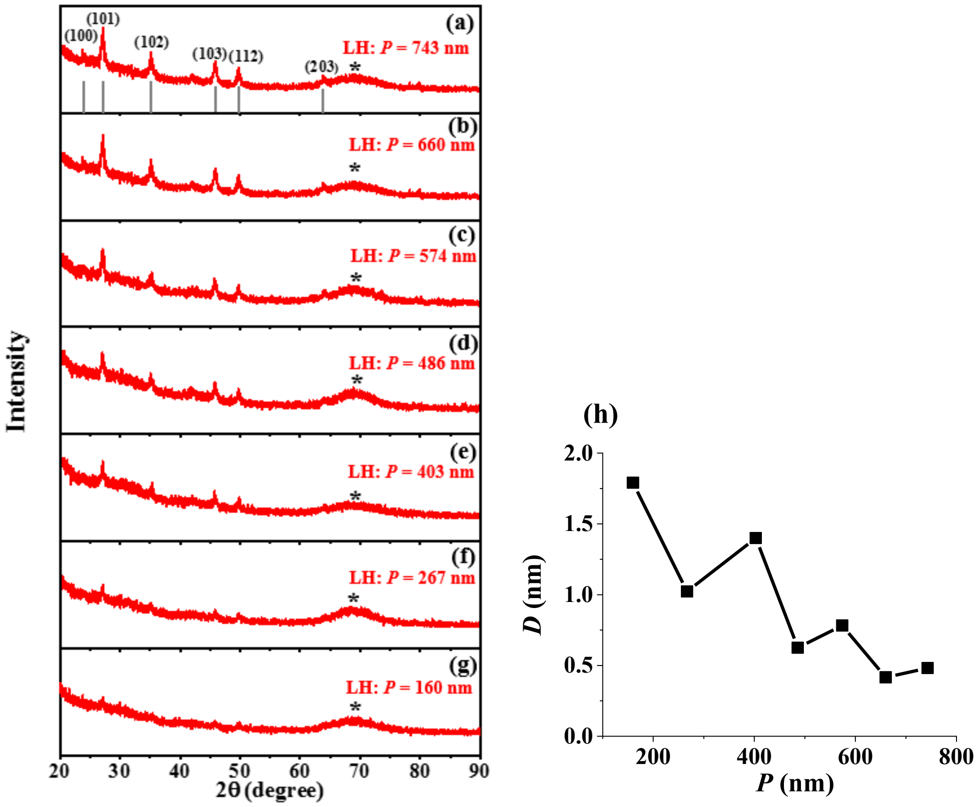

Figure S3. XRD spectra of LH-CdSe NHs deposited on silicon wafers as function of *P*: (a) 743 nm, (b) 660 nm, (c) 574 nm, (d) 486 nm, (e) 403 nm, (f) 267 nm, and (g) 160 nm. The XRD peaks of silicon wafers are marked with asterisks. (h) Plot of the average sizes of grain domains (*D*) of the CdSe NHs as a function of *P*.

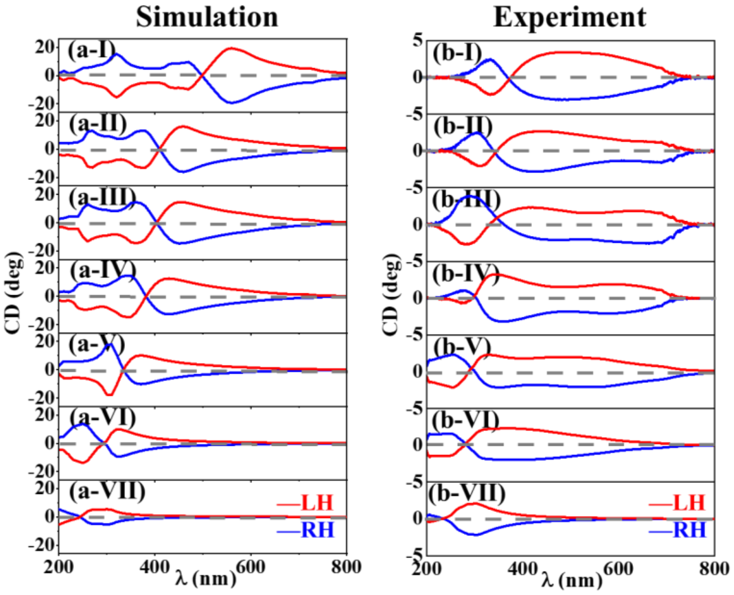

Figure S4. Comparison of (a) the simulated and (b) experimentally monitored CD spectra of the arrays of one-turn CdSe NHs, with a *P* of (a-I) 730 nm, (a-II) 590 nm, (a-III) 520 nm, (a-IV) 460 nm, (a-V) 410 nm, (a-VI) 300 nm, and (a-VII) 200 nm for both LH and RH; (b-I) 743 and 754 nm, (b-II) 660 and 611 nm, (b-III) 574 and 564 nm, (b-IV) 486 and 462 nm, (b-V) 403 and 392 nm, (b-VI) 267 and 301 nm, (b-VII) 160 and 163 nm, for LH and RH, respectively. LH: red lines; RH: blue lines. The other structural parameters for the simulation are summarized in **Table S2**.

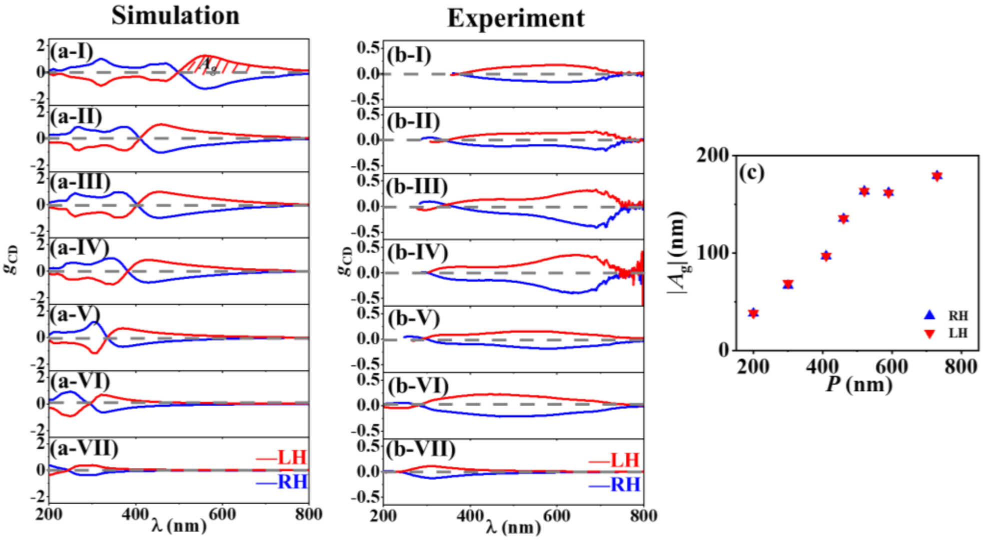

Figure S5. Comparison of (a) the simulated and (b) experimentally monitored g_{CD} spectra of the arrays of one-turn CdSe NHs, with a *P* of (a-I) 730 nm, (a-II) 590 nm, (a-III) 520 nm, (a-IV) 460 nm, (a-V) 410 nm, (a-VI) 300 nm, and (a-VII) 200 nm for both LH and RH; (b-I) 743 and 754 nm, (b-II) 660 and 611 nm, (b-III) 574 and 564 nm, (b-IV) 486 and 462 nm, (b-V) 403 and 392 nm, (b-VI) 267 and 301 nm, (b-VII) 160 and

163 nm, for LH and RH, respectively. LH: red lines; RH: blue lines. The other structural parameters for the simulation are summarized in **Table S2**. The integrated area of a g_{CD} peak in the visible region (A_{g}) is marked in (a-I). (c) Plots of A_{g} versus P. LH: red symbols; RH: blue symbols.

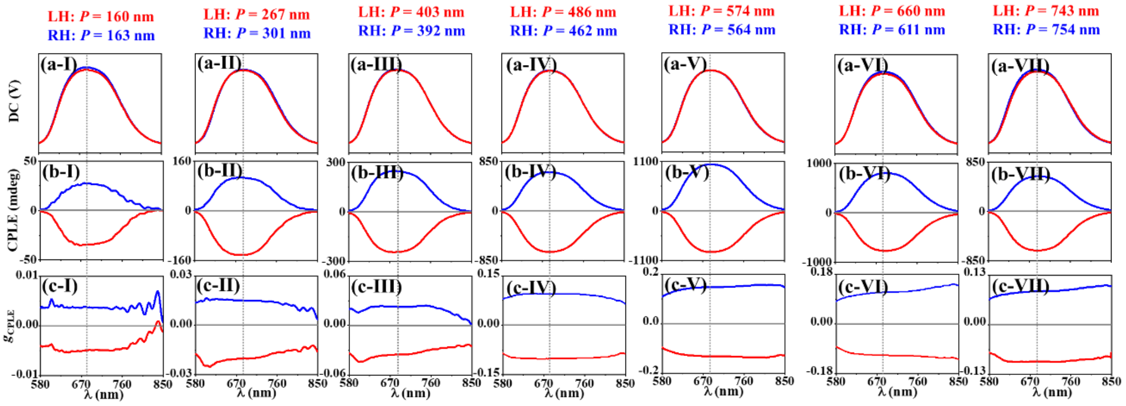

Figure S6. CPLE of one-turn CdSe NHs characterized with (a) photoluminescence, (b) CPLE, and (c) g_{CPLE} spectra. The CdSe NHs have a P of (I) 160 and 163 nm, (II) 267 and 301 nm, (III) 403 and 392 nm, (IV) 486 and 462 nm, (V) 574 and 564 nm, (VI) 660 and 611 nm, (VII) 743 and 754 nm, for LH (red lines) and RH (blue lines) CdSe NHs, respectively. The parameters shown in Figure 3(d-f) are monitored at the wavelength marked by grey dashed lines.

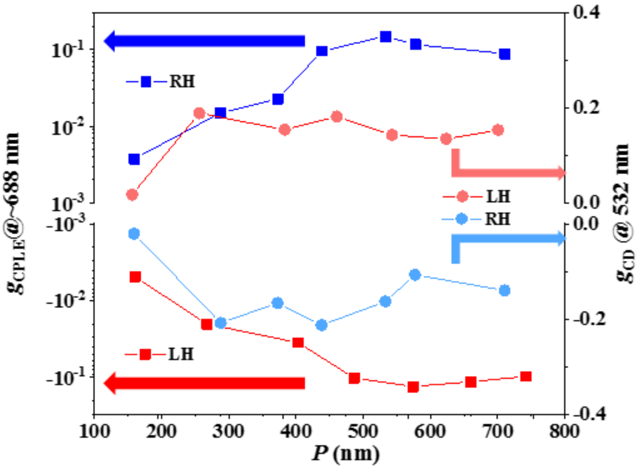

Figure S7. Plots of g_{CPLE} (measured at a wavelength of ≈688 nm, as shown in Figure 3e) and g_{CD} (measured at an incident wavelength of 532 nm, Figure S1c) versus P of one-turn CdSe NHs.

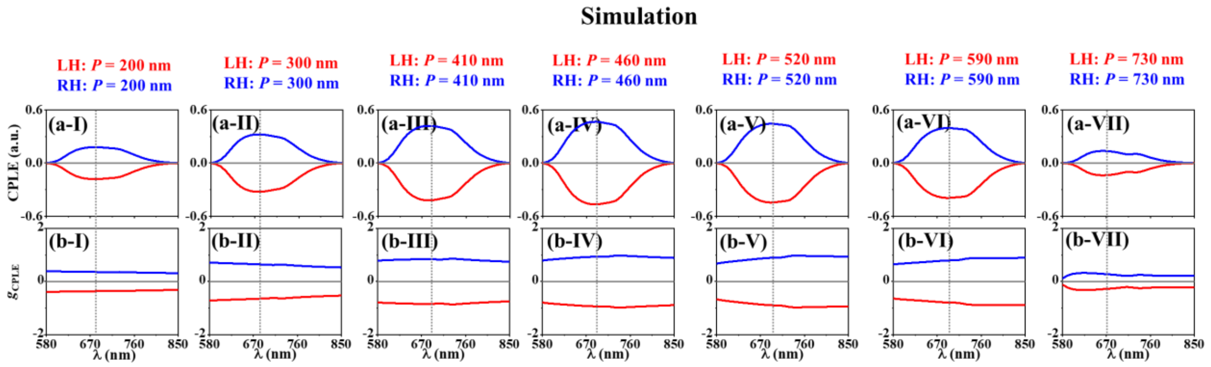

Figure S8. Simulation of (a) CPLE and (b) g_{CPLE} spectra for one-turn CdSe NHs, with a P of (I) 200 nm, (II) 300 nm, (III) 410 nm, (IV) 460 nm, (V) 520 nm, (VI) 590 nm, and (VII) 730 nm. LH: red lines; RH: blue lines.

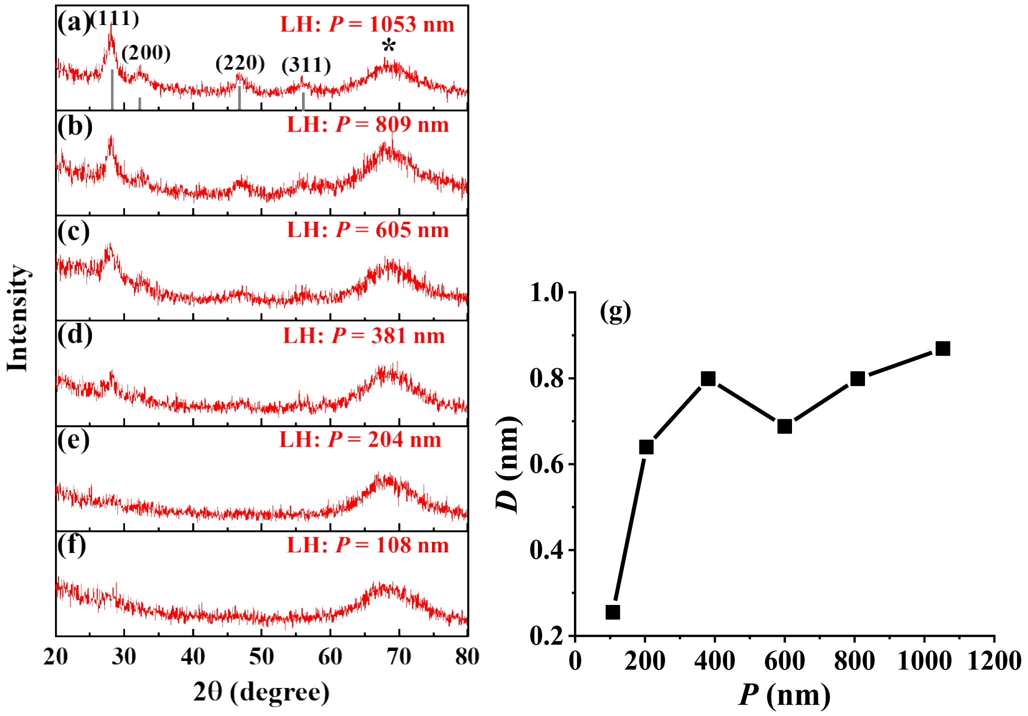

Figure S9. XRD spectra of LH-ceria NHs deposited on silicon wafers as s function of P: (a) 1,053 nm, (b) 809 nm, (c) 605 nm, (d) 381 nm, (e) 204 nm, and (f) 108 nm. The XRD peaks of silicon wafers are marked with an asterisk. (g) Plot of the average D values of the ceria NHs as a function of P.

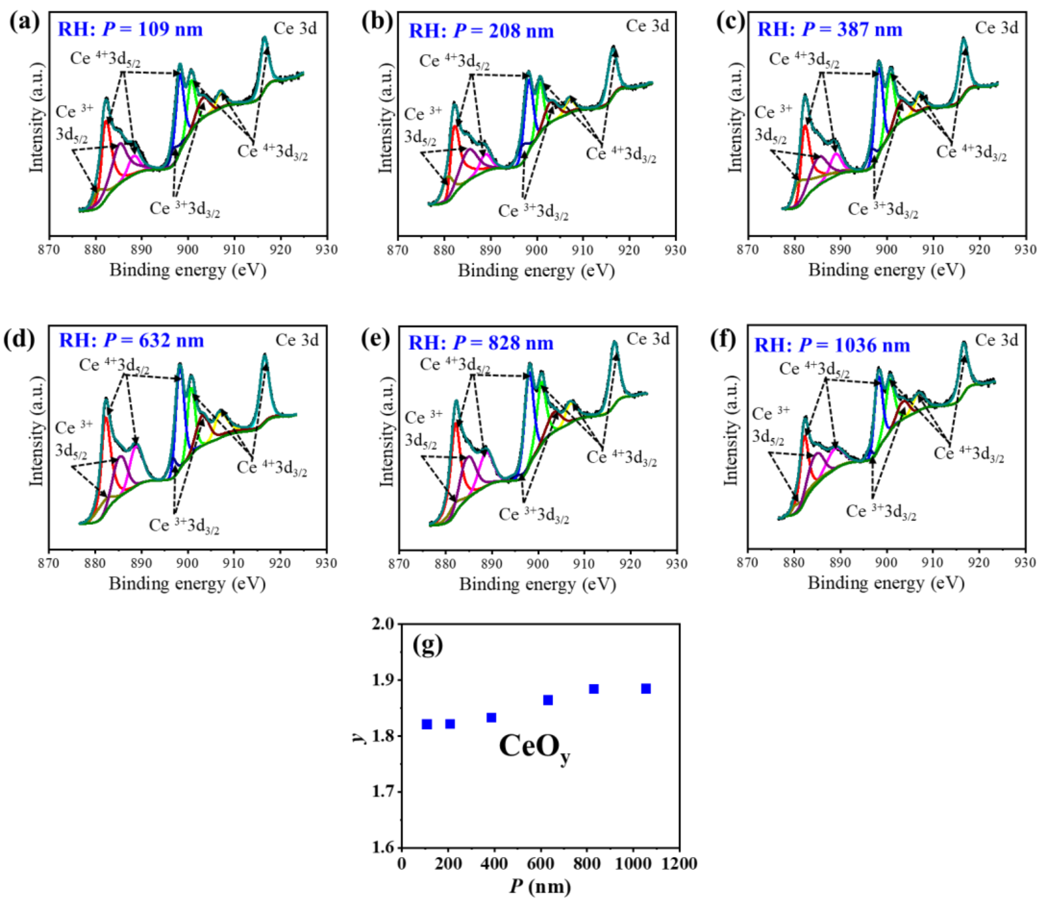

Figure S10. XPS spectra of the RH-ceria NHs: Ce 3d. The RH-ceria NHs have a *P* of (a) 109 nm, (b) 208 nm, (c) 387 nm, (d) 632 nm, (e) 828 nm, and (f) 1,036 nm. The Ce 3d spectra, composed of Ce $3d_{5/2}$ and Ce $3d_{3/2}$, were fitted with a set of peaks. The peaks at 884.28 eV and 902.09 eV are ascribed to Ce^{3+}, indicating the formation of oxygen vacancies at the helical surfaces. The other Ce 3d peaks at 882.58 eV, 889.11 eV, 898.46 eV, 901.04 eV, 907.80 eV and 917.09 eV are attributed to Ce^{4+}. The ratio of the integrated areas of the Ce^{3+} peaks to those of the Ce^{4+} peaks was calculated to evaluate (g) the stoichiometric *y* in the as-deposited one-turn RH-CeO_y NHs, as a function of *P*.

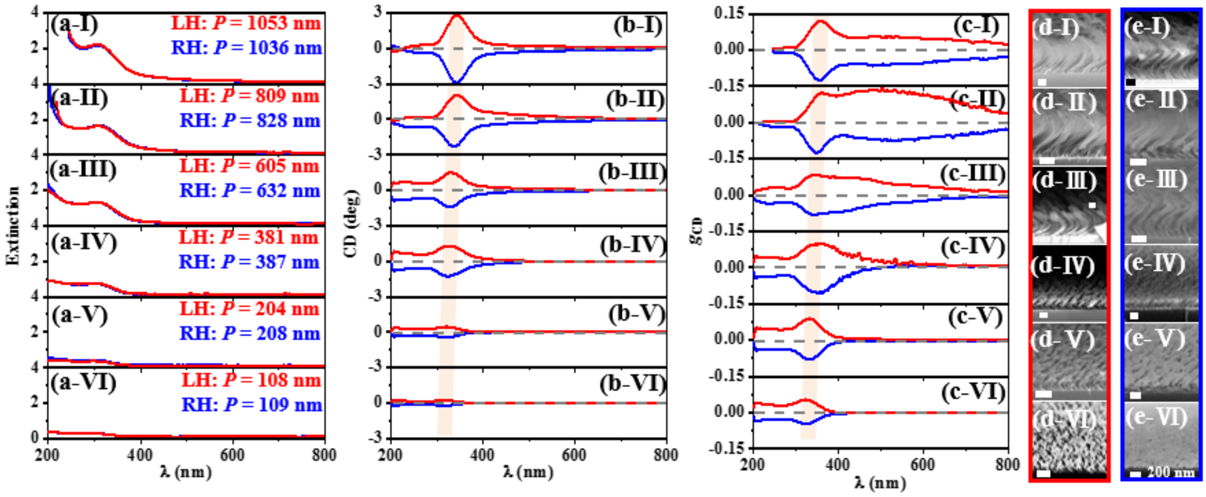

Figure S11. GLAD of one-turn ceria NHs as a function of *P*: (I) 1053 and 1036 nm, (II) 809 and 828 nm, (III) 605 and 632 nm, (IV) 381 and 387 nm, (V) 204 and 208 nm, (VI) 108 and 109 nm, for LH- and RH-ceria NHs, respectively. UV–visible-NIR spectra of (a) extinction, (b) CD and (c) g_{CD} (LH: red lines; RH: blue lines). (b, c) The CD and g_{CD} peaks in the UV region appears to have a red shift with an increase of *P*, marked by light red backgrounds. SEM tilted images of (d) LH- and (e) RH-ceria NHs (scale bars: 200 nm).

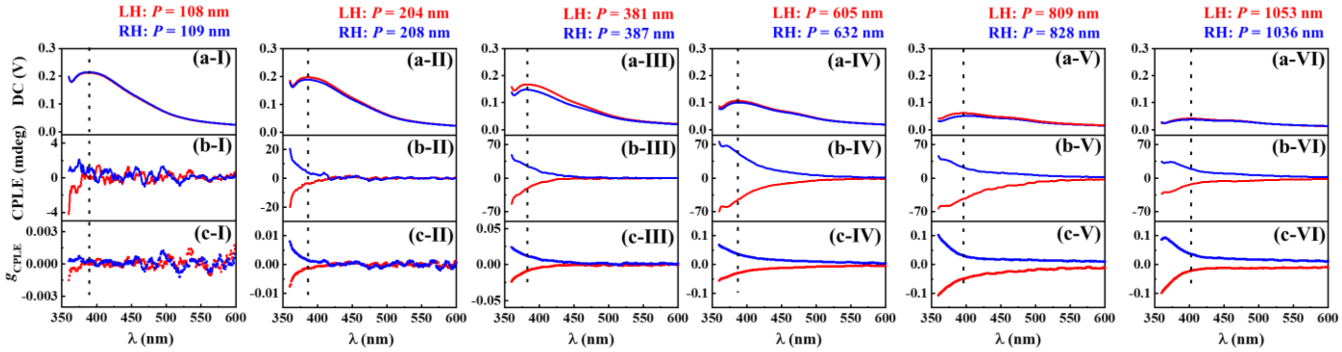

Figure S12. CPLE of one-turn ceria NHs characterized with (a) photoluminescence, (b) CPLE, and (c) g_{CPLE} spectra. The ceria NHs have a *P* of (I) 108 and 109 nm, (II) 204 and 208 nm, (III) 381 and 387 nm, (IV) 605 and 632 nm, (V) 809 and 828 nm, (VI) 1053 and 1036 nm, for LH (red lines) and RH (blue lines) NHs, respectively. The parameters shown in Figure 6d-6g are monitored at the wavelength marked by black dashed lines.

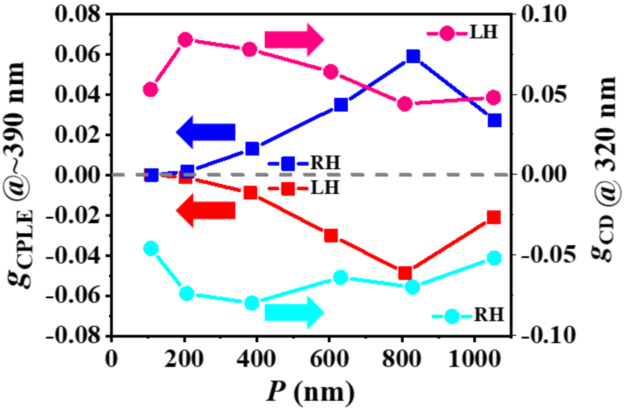

Figure S13. Plots of g_{CPLE} (measured at a wavelength of ≈390 nm, as shown in Figure 6e and 6f) and g_{CD} (measured at an incident wavelength of 320 nm, Figure S11c) versus P of the one-turn ceria NHs.

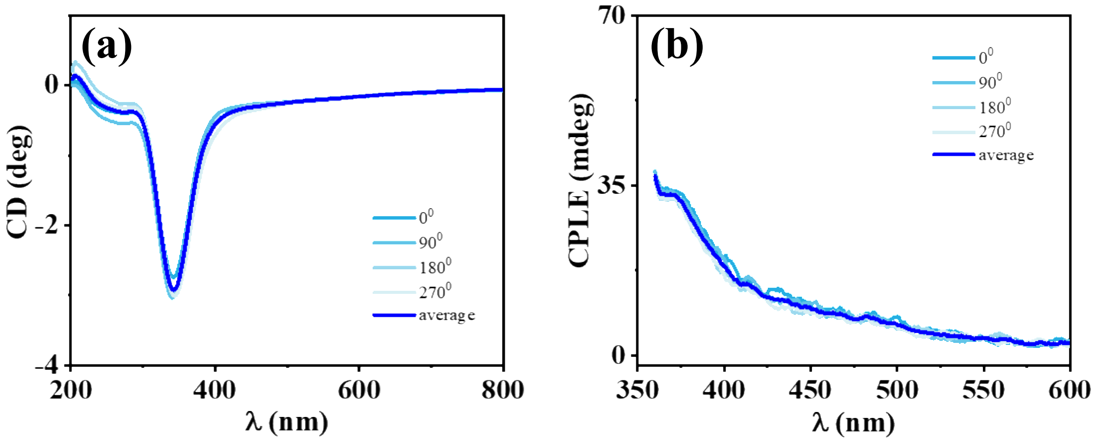

Figure S14. UV-visible (a) CD and (b) CPLE spectra of RH-ceria NHs with a P of 1036 nm, monitored at a sample rotation angle of 0^0, 90^0, 180^0 and 270^0.

Table S1. Comparison of the $|g_{CD}|$ values of chiral CdSe nanostructures.

Chiral CdSe nanomaterials	Highest $|g_{CD}|$ values	References
**CdSe NHs**	**0.4**	**this work**
Chiral stacking of CdSe/CdS alloy nanorods	0.05	[S1]
Chiral self-assembly of CdSe@CdS (core@shell) nanorods	0.02	[S2]
CdSe/CdS alloy quantum dots (QDs) modified with L-penicillamine	1.7×10^{-3}	[S3]
CdSe QDs modified with chiral carboxylic Acids	7.0×10^{-4}	[S4]
CdSe quantum rods modified with L-/D-cysteine	5.0×10^{-4}	[S5]
CdSe nanocrystals modified with L-/D-cysteine	4.0×10^{-4}	[S6]
CdSe QDs modified with N-acetyl-L-cysteine	1.3×10^{-4}	[S7]
CdSe/CdS alloy QDs modified with L-/D-cysteine	6.0×10^{-5}	[S8]
CdSe/CdS alloy QDs modified with L-/D-cysteine	4.0×10^{-5}	[S9]

Table S2. Structure parameters of the one-turn LH- and RH-CdSe NHs used in the numerical simulations (as shown in Figure 2a and 2d-I), according to the structural characterizations with the SEM images. *P*: the helical pitch; *D*: the coil diameter of NHs; d_b: the bottom wire diameter of NHs; d_t: the top wire diameter of NHs; *a*: the period in a NH array.

CdSe NHs	*P* (nm)	*D* (nm)	d_b (nm)	d_t (nm)	*a* (nm)
VII	200	110	20	50	130
VI	300	190	30	60	210
V	410	260	40	70	280
IV	460	300	40	100	320
III	520	320	50	110	340
II	590	330	50	110	350
I	730	410	50	140	430

Table S3. Comparison of the $|g_{CPLE}|$ values of chiral CdSe nanostructures.

**Chiral CdSe nanomaterials**	**Highest $|g_{CPLE}|$ values**	**References**
CdSe/ZnS QDs assembled on chiral cholesteric	1.6	[S10]
CdSe@CdS quantum rods assembled to chiral ordered multilayer structures	0.8	[S11]
Chiral cellulose nanocrystals doped with CdSe/CdS QDs	0.45	[S12]
**CdSe NHs**	**0.15**	**this work**
Chiral stacking of CdSe/CdS alloy nanorods	0.0997	[S1]
CdSe/CdS NRs assembled on chiral PDI Monomers	0.053	[S13]
CdSe QDs induced by L- and D-cysteine	0.003 (L-)<br>0.004 (D-)	[S14]
CdSe/ZnS QDs induced by nucleotide amino acids	2.0×10^{-3}	[S15]
CdSe/CdS nanoplatelets L- and D-cysteine	5.29×10^{-4}	[S16]
CdSe-dot/CdS-rods induced by L- and D-cysteine	4.66×10^{-4}	[S17]

References

S1. Chen, L.; Hao, C.; Cai, J.; Chen, C.; Ma, W.; Xu, C.; Xu, L.; Kuang, H. Chiral Self-Assembled Film from Semiconductor Nanorods with Ultra-Strong Circularly Polarized Luminescence. *Angew. Chem. Int. Ed.* **2021,** *60*, 26276-26280.

S2. Duan, T.; Ai, J.; Cui, X.; Feng, X.; Duan, Y.; Han, L.; Jiang, J.; Che, S. Spontaneous chiral self-assembly of CdSe@CdS nanorods.*Chem* **2021,** *7*, 2695-2707.

S3. Moshe, A. B.; Markovich, G. Chiral Ligand-Induced Circular Dichroism in Excitonic Absorption of Colloidal Quantum Dots. *Isr. J. Chem.* **2012,** *52*, 1104-1110.

S4. Puri, M.; Ferry, V. E. Circular dichroism of CdSe nanocrystals bound by chiral carboxylic acids. *ACS Nano* **2017,** *11*, 12240-12246.

S5. Gao, X.; Zhang, X.; Deng, K.; Han, B.; Zhao, L.; Wu, M.; Shi, L.; Lv, J.; Tang, Z. Excitonic circular dichroism of chiral quantum rods. *J. Am. Chem. Soc.* **2017,** *139*, 8734-8739.

S6. Shao, X.; Wu, Y.; Jiang, S.; Li, B.; Zhang, T.; Yan, Y. Chiral 3D CdSe Nanotetrapods. *Inorg. Chem.* **2020,** *59*, 14382-14388.

S7. Choi, J. K.; Haynie, B. E.; Tohgha, U.; Pap, L.; Elliott, K. W.; Leonard, B. M.; Dzyuba, S. V.; Varga, K.; Kubelka, J.; Balaz, M. Chirality inversion of CdSe and CdS quantum dots without changing the stereochemistry of the capping ligand. *ACS Nano* **2016,** *10*, 3809-3815.

S8. Kuznetsova, V. A.; Mates-Torres, E.; Prochukhan, N.; Marcastel, M.; Purcell-Milton, F.; O'Brien, J.; Visheratina, A. K.; Martinez-Carmona, M.; Gromova, Y.; Garcia-Melchor, M.; Gun'ko, Y. K. Effect of chiral ligand concentration and binding mode on chiroptical activity of CdSe/CdS quantum dots. *ACS Nano* **2019,** *13*, 13560-13572.

S9. Purcell-Milton, F.; Visheratina, A. K.; Kuznetsova, V. A.; Ryan, A.; Orlova, A. O.; Gun'ko, Y. K. Impact of shell thickness on photoluminescence and optical activity in chiral CdSe/CdS core/shell quantum dots. *ACS Nano* **2017,** *11*, 9207-9214.

S10. Bobrovsky, A.; Mochalov, K.; Oleinikov, V.; Sukhanova, A.; Prudnikau, A.; Artemyev, M.; Shibaev, V.; Nabiev, I. Optically and electrically controlled circularly polarized emission from cholesteric liquid crystal materials doped with semiconductor quantum dots. *Adv. Mater.* **2012,** *24*, 6216-6222.

S11. Lv, J.; Yang, X.; Tang, Z. Rational Design of all-inorganic Assemblies with Bright Circularly Polarized luminescence. *Adv. Mater.* **2022**, *35*, 2209539.

S12. Shi, Y.; Zhou, Z.; Miao, X.; Liu, Y. J.; Fan, Q.; Wang, K.; Luo, D.; Sun, X. W. Circularly polarized luminescence from semiconductor quantum rods templated by self-assembled cellulose nanocrystals. *J. Mater. Chem. C* **2020,** *8*, 1048-1053.

S13. Liu, R.; Feng, Z.; Cheng, C.; Li, H.; Liu, J.; Wei, J.; Yang, Z. Active Regulation of Supramolecular Chirality through Integration of CdSe/CdS Nanorods for Strong and

Tunable Circular Polarized Luminescence. *J. Am. Chem. Soc.* **2022,** *144*, 2333-2342.
S14. Tohgha, U., Kirandeep K. Deol, Ashlin G. Porter, Samuel G. Bartko, Jung Kyu Choi, Brian M. Leonard, Krisztina Varga, Jan Kubelka, Gilles Muller, and Milan Balaz. Ligand induced circular dichroism and circularly polarized luminescence in CdSe quantum dots. *ACS Nano* **2013,** *7*, 11094-11102.
S15. Wen, X.; Fan, H.; Jing, L.; Deng, M.; Huang, X.; Jiao, T.; Zhang, L.; Liu, M. Competitive induction of circularly polarized luminescence of CdSe/ZnS quantum dots in a nucleotide-amino acid hydrogel. *Mater. Adv.* **2022,** *3*, 682-688.
S16. Hao, J.; Zhao, F.; Wang, Q.; Lin, J.; Chen, P.; Li, J.; Zhang, D.; Chen, M.; Liu, P.; Delville, M. H.; He, T.; Cheng, J.; Li, Y. Optically Active CdSe/CdS Nanoplatelets Exhibiting Both Circular Dichroism and Circularly Polarized Luminescence. *Adv. Opt. Mater.* **2021,** *9*, 2011142.
S17. Cheng, J.; Hao, J.; Liu, H.; Li, J.; Li, J.; Zhu, X.; Lin, X.; Wang, K.; He, T. Optically active CdSe-dot/CdS-rod nanocrystals with induced chirality and circularly polarized luminescence. *ACS Nano* **2018,** *12*, 5341-5350.
